# Mechanisms of mutational robustness in transcriptional regulation

**DOI:** 10.3389/fgene.2015.00322

**Published:** 2015-10-27

**Authors:** Joshua L. Payne, Andreas Wagner

**Affiliations:** ^1^Institute of Evolutionary Biology and Environmental Studies, University of ZurichZurich, Switzerland; ^2^Swiss Institute of BioinformaticsLausanne, Switzerland; ^3^The Santa Fe InstituteSanta Fe, NM, USA

**Keywords:** homotypic clusters, redundancy, regulatory networks, shadow enhancers, transcription factor binding sites

## Abstract

Robustness is the invariance of a phenotype in the face of environmental or genetic change. The phenotypes produced by transcriptional regulatory circuits are gene expression patterns that are to some extent robust to mutations. Here we review several causes of this robustness. They include robustness of individual transcription factor binding sites, homotypic clusters of such sites, redundant enhancers, transcription factors, redundant transcription factors, and the wiring of transcriptional regulatory circuits. Such robustness can either be an adaptation by itself, a byproduct of other adaptations, or the result of biophysical principles and non-adaptive forces of genome evolution. The potential consequences of such robustness include complex regulatory network topologies that arise through neutral evolution, as well as cryptic variation, i.e., genotypic divergence without phenotypic divergence. On the longest evolutionary timescales, the robustness of transcriptional regulation has helped shape life as we know it, by facilitating evolutionary innovations that helped organisms such as flowering plants and vertebrates diversify.

## 1. Introduction

Robustness is the invariance of a phenotype in the face of environmental or genetic change. The phenotypes of living systems exhibit robustness at multiple scales of organization, ranging from the structural properties of macromolecules (Bloom et al., 2005; Wagner, 2008) to the preferred carbon sources of entire metabolisms (Samal et al., 2010). An immense body of work has focused on elucidating the mechanisms of robustness in living systems (reviewed in de Visser et al., 2003; Kitano, 2004; Stelling et al., 2004; Wagner, 2005; Masel and Siegal, 2009). Here we highlight a subset of this work, specifically those studies that have addressed the mechanisms of mutational robustness in transcriptional regulation.

Transcriptional regulation is fundamental to the control of gene expression. It allows cells to respond to environmental signals (Ptashne and Gann, 2002), such as hormones or sugars, and it drives fundamental behavioral and developmental processes, such as mating in yeast (Tsong et al., 2006) and embryonic patterning in fruit flies (Lawrence, 1992). Transcriptional regulation is largely carried out by transcription factors (TFs), proteins that bind short DNA sequences—TF binding sites—in the promoters or enhancers of genes. Such binding may induce or repress gene expression by promoting or inhibiting the recruitment of RNA polymerase. Given the fundamental importance of when and where genes are expressed, it is crucial that transcriptional regulation is robust to perturbation.

Genetic perturbations that may affect transcriptional regulation occur in both *cis* and in *trans*. They include point mutations in TF binding sites, which may impact transcriptional regulation by changing the affinity of a binding site for its cognate TF. They also include the insertion or deletion of large segments of DNA within promoters or enhancers, which may add or remove one or more regulatory interactions from a regulatory circuit. And they include changes to the amino acid sequence of the activation or DNA binding domains of a TF, which may alter the entire binding repertoire of the TF. Such perturbations can be deleterious, as shown by the numerous disease-associated mutations within gene regulatory regions and within genes that encode TFs (Vaquerizas et al., 2009; Maurano et al., 2012; Lee and Young, 2013).

**Figure 1:**
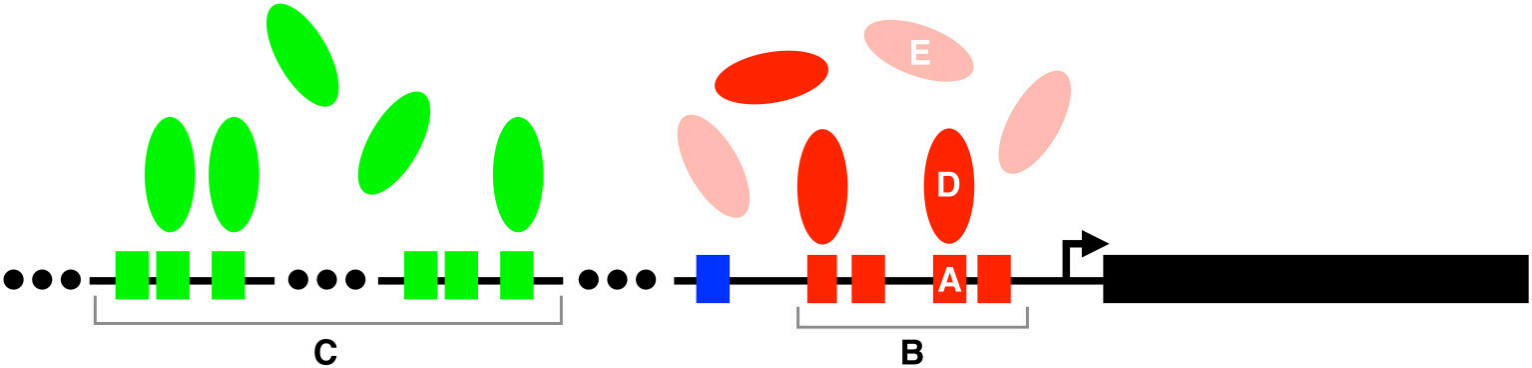
**Mechanisms of mutational robustness in transcriptional regulation**. Robustness can be conferred by (A) individual transcription factor binding sites, (B) homotypic clusters of such sites, (C) redundant enhancers, (D) individual transcription factors, and (E) redundant transcription factors. Small colored boxes represent transcription factor binding sites, ellipsoids represent transcription factors, and the arrow represents the transcription start site of the gene indicated by the large black rectangle. The lightly shaded ellipses in (E) represent paralogs of the transcription factors (red ellipses) in (D). Both the red and green transcription factors regulate the expression of the black gene. These regulatory interactions are part of a larger regulatory network, whose structural properties can also influence the robustness of transcriptional regulation.

Transcriptional regulation is not only subject to a litany of genetic insults, it is also remarkably robust to these insults (Weirauch and Hughes, 2010). Gene expression phenotypes are often insensitive to mutations in TF binding sites (Kasowski et al., 2010; Kwasnieski et al., 2012), to the turnover of regulatory control from one TF to another (Ludwig et al., 2000; Odom et al., 2007), to variation in gene expression levels (Garfield et al., 2013), and even to the rewiring of entire transcriptional regulatory circuits (Tsong et al., 2006; Isalan et al., 2008; Swanson et al., 2011). Here, we review the mechanisms that underlie this mutational robustness (Figure 1). Reviews of the equally important topic of robustness to environmental perturbations can be found elsewhere (Eldar et al., 2004; Alon, 2007; Macneil and Walhout, 2011; Silva-Rocha and de Lorenzo, 2010), as can primary literature on the contribution of post-transcriptional regulation to robust gene expression (McManus et al., 2014).

## 2. Mechanisms of robustness

### 2.1. Transcription factor binding sites

TF binding sites are short DNA sequences (6–12 base pairs) that bind TFs to regulate gene expression. On the one hand, mutations in TF binding sites can be deleterious, as shown by their involvement in human disease (Pomerantz et al., 2009; Musunuru et al., 2010; Harismendy et al., 2011), including cancer (Khurana et al., 2013; Weinhold et al., 2014; Katainen et al., 2015; Melton et al., 2015). For instance, of 2931 disease-associated single nucleotide polymorphisms located within regulatory DNA, 93.2% fall within TF binding sites (Maurano et al., 2012). On the other hand, cross-species comparisons of regulatory regions often uncover variation in TF binding sites without obvious differences in the gene expression patterns that are driven by these sites (Ludwig et al., 2000; Odom et al., 2007). In addition, within-species variation in TF binding sites is common (Garfield et al., 2012; Spivakov et al., 2012; Arbiza et al., 2013; Khurana et al., 2013; Zheng et al., 2011), and such inter-individual differences often do not affect the expression level of target genes (Kasowski et al., 2010; Zheng et al., 2010).

The simplest cause of such mutational robustness is that individual binding sites are themselves robust to mutation. That is, they can often tolerate mutations without losing the ability to bind their cognate TFs. This results from two properties of TFs: (1) They typically bind dozens, if not hundreds of distinct DNA sequences (Sengupta et al., 2002; Berger et al., 2006; Badis et al., 2009; Wong et al., 2013) and (2) these sequences are almost always organized as large *genotype networks* in the space of all possible binding sites (Payne and Wagner, 2014). In such a genotype network, nodes represent DNA sequences that bind a particular TF and edges connect nodes if their corresponding sequences differ by a single small DNA mutation. Genotype networks confer robustness, because a mutation to any site in a TF's binding site repertoire is likely to yield another site that is also in the repertoire, thus preserving binding. Moreover, the binding affinities of neighboring sites in a genotype network are strongly correlated, indicating that a site's affinity for a TF is also robust to mutation. This is important, because mutations that affect binding affinity may impact the expression of a TF's target genes (Kasowski et al., 2010; Shultzaberger et al., 2010; Sharon et al., 2012). In addition, it is worth highlighting that the very short length of TF binding sites itself confers mutational robustness: Even though longer sites may offer greater specificity, they are also more susceptible to mutational disruption (Stewart et al., 2012).

### 2.2. Homotypic clusters of transcription factor binding sites

Regulatory regions often contain multiple binding sites for the same TF (Johnson et al., 1979; Giniger and Ptashne, 1988; Carey et al., 1990; Thanos and Maniatis, 1995; Wasserman and Fickett, 1998; Krivan and Wasserman, 2001; Berman et al., 2002; Ezer et al., 2014). Such *homotypic clusters* of binding sites are common in both prokaryotic and eukaryotic organisms, including bacteria (Gama-Castro et al., 2011), fruit flies (Lifanov et al., 2003), and humans (Gotea et al., 2010). For example, in humans, 62% of promoters and roughly 40% of 487 experimentally-validated developmental enhancers contain such clusters (Gotea et al., 2010). The benefits of homotypic clusters include threshold-dependent (Lebrecht et al., 2005) and graded (Giogetti et al., 2010) transcriptional responses to input signals.

An additional benefit of homotypic clusters is mutational robustness. Experiments with high-throughput promoter screens show that increasing the number of binding sites within a homotypic cluster has a saturating effect on gene expression, such that increasing the number of sites beyond a threshold results in no further impact on gene expression (Sharon et al., 2012; Smith et al., 2013). This apparent redundancy of a subset of a cluster's binding sites can provide robustness to mutation. For example, the promoter of the mouse HTF9 genes contains a homotypic cluster of binding sites for the TF Sp1, and deletion of all but one of these sites has no effect on the expression of HTF9 genes (Somma et al., 1991). Similarly, mutations in a binding site of the human TF PU.1 are less likely to impact gene expression if a second, non-mutated site is nearby (Kilpinen et al., 2013). This finding echoes earlier observations made in an analysis of polymorphic TF binding sites in *Drosophila melanogaster*, which found that sites were more likely to tolerate deleterious mutations if they were located nearby other sites for the same TF (Spivakov et al., 2012).

### 2.3. Redundant enhancers

Enhancers are DNA sequences (50–1500 base pair) that bind one or more TFs to activate the transcription of genes, often in a cell-specific manner (Banerji et al., 1981; de Villiers et al., 1982; Gillies et al., 1983; Small et al., 1996; Levine et al., 2014; Shlyueva et al., 2014). Enhancers often target genes across long chromosomal distances, but typically within well-defined structural units called topologically associating domains (Dixon et al., 2012). Many genes are regulated by more than one enhancer, as exemplified by the gap genes in *Drosophila*, which control anterior-posterior patterning in the developing embryo. For example, the gap genes *hunchback, Kruppel*, and *knirps* are each regulated by two distinct enhancers that work together to produce bands of gene expression in the presumptive head, thorax, and abdomen (Perry et al., 2011). More generally, a genome-wide analysis of enhancer activity in *Drosophila* S2 cells found that 434 genes are regulated by at least two enhancers, and 203 of these genes are regulated by more than five enhancers (Arnold et al., 2013). For many genes, all of the gene's enhancers are necessary to drive appropriate expression. For example, both of the enhancers that regulate the gap gene *hunchback* are necessary to ensure the gene's correct expression in the developing embryo (Perry et al., 2011). In some genes, however, enhancers appear to be functionally redundant: Under normal growth conditions, only one of a gene's multiple enhancers are necessary to drive correct expression (Frankel et al., 2010; Perry et al., 2010).

Redundant enhancers—sometimes referred to as *shadow enhancers* (Hong et al., 2008)—provide not only robustness to environmental perturbations (Frankel et al., 2010; Perry et al., 2010), but also robustness to mutations. This is because deletion of one enhancer is often insufficient to disrupt normal gene expression, even if the enhancers are only partially redundant. For example, the *Drosophila* gene *snail*—a key determinant of dorsal-ventral patterning—is regulated by two enhancers, and deletion of either of these enhancers does not alter the gene's expression pattern in the presumptive mesoderm under normal growth conditions (Perry et al., 2010). Redundant enhancers can also provide robustness to mutations that affect the expression level of their cognate TFs (Frankel et al., 2010; Perry et al., 2010). For example, the two enhancers of *snail* drive a normal pattern of expression upon reduction of the expression level of Dorsal, an activator of *snail*, whereas deletion of one of these enhancers yields erratic patterns of *snail* expression in response to this genetic perturbation (Perry et al., 2010).

We note that shadow enhancers do not always provide mutational robustness. For example, the *Drosophila* gene *shavenbaby* is regulated by three primary enhancers and two shadow enhancers (Frankel et al., 2010). While the shadow enhancers are not necessary to drive the gene's epidermal expression pattern under normal growth conditions, their presence does not compensate for the inactivation of any one of the three primary enhancers (McGregor et al., 2007).

### 2.4. Transcription factors

Transcription factors are also to some extent robust to mutations, including those that change the amino acid sequence of the protein's DNA binding domain. There are at least two causes of this robustness. First, amino acid substitutions in a TF's DNA binding domain may have little or no effect on the TF's binding specificity. For example, the human helix-loop-helix transcription factor Max contacts DNA at five residues, and amino acid substitutions in three of these residues have no effect on binding specificity (Maerkl and Quake, 2009). Second, transcription factors often bind DNA cooperatively, and the presence of cofactors may ameliorate the effects of amino acid substitutions that impair binding specificity. For example, the binding specificity of Matα1, a regulator of cell-type specification in ascomycete fungi, has diverged so extensively among *S. cerevisiae* and *C. albicans* that the sequences recognized by these proteins appear unrelated by bioinformatic criteria (Baker et al., 2011). Nonetheless, Matα1 controls the same set of core genes in these two species, because its recognition sequences evolved along with it. This was most likely facilitated by a protein-protein interaction with Mcm1, which is conserved among *S. cerevisiae* and *C. albicans*, and may have helped stabilize Matα1 while its interaction with DNA slowly changed.

Despite these examples, it should be emphasized that mutations in a transcription factor's DNA binding domain often do affect binding specificity and that cofactors cannot always compensate for such changes. Because transcription factors typically regulate the expression of multiple genes, such mutations are often deleterious. This is demonstrated both by the common implication of such mutations in disease (Lee and Young, 2013) and by the high level of conservation of one-to-one transcription factor orthologs across highly diverse species (Nitta et al., 2015).

### 2.5. Redundant transcription factors

Gene duplication, which creates paralogous genes within the same genome, is a driving force in evolution. In eukaryotes, for instance, gene duplicates are estimated to arise at a rate of 0.01 per gene per million years (Lynch and Conery, 2000), and between 30 and 65 percent of a typical eukaryote's genes have paralogs (Zhang, 2003). Because gene duplicates are often functionally redundant at their time of origin, it is possible that they play compensatory roles, acting as a backup if one of the paralogs is functionally compromised. This possibility has led to a large body of research on redundant genes as a source of mutational robustness (e.g., Conant and Wagner, 2003; Gu et al., 2003).

Gene duplication has played an important role in the evolution of transcriptional regulatory systems. For example, an estimated 68% of TFs in yeast (Teichmann and Babu, 2003) and 73% of TFs in *Escherichia coli* (Madan Babu and Teichmann, 2003) are the result of gene duplication. Many of these paralogous transcription factors appear fully or partially redundant in function, because they recognize the same sets of binding sites *in vitro* (Weirauch et al., 2014) and bind to some of the same genomic regions *in vivo*. For example, genome-wide binding profiles of three ETS TFs in human T cells revealed that nearly 10% of 17,000 promoters bound more than two of the three TFs, and probably at the same binding site (Hollenhorst et al., 2007). A broader view of redundant TFs is provided by enhanced yeast one-hybrid assays (Reece-Hoyes et al., 2011), which have facilitated a test of nearly 400,000 putative binding events among 1086 human TFs and 360 enhancers (Fuxman Bass et al., 2015). This analysis found that human enhancers often bind multiple TFs that typically belong to the same TF family. Moreover, the greater the number of enhancers that a pair of TFs shares, the more likely it is that these factors are coexpressed, and the less likely it is that each factor is essential for viability (Fuxman Bass et al., 2015), providing additional support for their compensatory roles. Indeed, even distant paralogs may compensate for one another, at least in part (Kafri et al., 2005; He and Zhang, 2006; Tischler et al., 2006).

### 2.6. Global topological properties of transcriptional regulatory networks

The transcriptional regulatory networks of organisms as different as bacteria and humans exhibit strikingly similar structural properties, including a heavy-tailed degree distribution, a modular organization, and non-random assortativity (Barabási and Oltvai, 2004; Boyle et al., 2014; Sorrells and Johnson, 2015). Each of these properties may confer mutational robustness in transcriptional regulation.

Many biological networks, including transcriptional regulatory networks, exhibit a heavy-tailed degree distribution (Aldana et al., 2007). Such networks are characterized by a preponderance of nodes with few connections and a small number of nodes with many connections. This topological property can endow a network with robustness to random gene deletion, because such deletions are more likely to affect low-degree nodes than high-degree nodes, and are therefore unlikely to disrupt the structure of a network (Albert et al., 2000). Simulations of model regulatory networks with heavy-tailed degree distributions show that such networks exhibit stable dynamical behavior over a broader range of parameter values than networks with a homogeneous degree distribution (Aldana and Cluzel, 2003). They are also more robust to both gene duplication (Aldana et al., 2007) and edge rewiring (Greenbury et al., 2010).

Transcriptional regulatory networks are modular. They can be decomposed into subnetworks of genes that are coregulated in response to different conditions and that are involved in distinct functions (Ihmels et al., 2002; Segal et al., 2003; Peter and Davidson, 2009). For example, an analysis of gene expression data in yeast uncovered 85 partially overlapping modules that participate in distinct cellular processes, including sporulation and rRNA processing (Ihmels et al., 2002). Similarly, the regulatory network controlling embryogenesis in the sea urchin has been decomposed into several modules that each perform distinct functions in patterning the pre-gastrular embryo, such as restricting gene expression to specific subdomains (Peter and Davidson, 2009). Such modularity may serve to contain damage, limiting the propagation of a mutation's effects to those genes that are also part of the module. For example, the yeast TF Ypl230w drives the expression of a module of hundreds of genes during entry to stationary phase. Analysis of differential gene expression upon deletion of Ypl230w found that differentially expressed genes were enriched within the module, indicating that the effect of the perturbation was largely contained (Segal et al., 2003). Similar observations have been made in simulations of model regulatory networks (Poblanno-Balp and Gershenson, 2011). It is therefore conceivable that modularity confers mutational robustness (Wagner et al., 2007), although in the context of transcriptional regulation, we currently have very little empirical evidence to support this possibility.

Assortativity is the propensity of nodes in a network to connect to other nodes with similar properties (Newman, 2002). For instance, in a network that is assortative with respect to the number of neighbors that a node (TF) has, nodes with many neighbors tend to connect to other nodes with many neighbors, and nodes with few neighbors tend to connect to nodes with few neighbors. Simulations of model transcriptional regulatory networks suggest that degree assortativity can confer robustness to mutations in regulatory regions (Pechenick et al., 2012) and to gene duplications (Pechenick et al., 2013). The transcriptional regulatory networks of 41 distinct human cell and tissue types exhibit such an assortativity signature (Pechenick et al., 2014), raising the possibility that this structural property confers robustness to transcriptional regulation in humans.

## 3. Origins of robustness

There are at least three possible origins of mutational robustness (de Visser et al., 2003): (1) Mutational robustness may itself be an adaptation to mutations, i.e., it may exist because it provides a selective advantage; (2) It may be a byproduct of other adaptations, such as environmental robustness; or, (3) It may be neither a direct adaptation nor an indirect by-product of an adaptation, and thus a non-adaptive result of biophysical principles or non-adaptive evolutionary forces.

The first, adaptive view can be traced to at least the early 1990s, when genetic studies first showed that many genes, including genes encoding TFs, are duplicated (Thomas, 1993). This observation raised the question whether such gene redundancy exists to protect genes against otherwise deleterious mutations, and lead to modeling work addressing this question (Clark, 1994; Nowak et al., 1997; Wagner, 1999, 2000; Lynch et al., 2001; O'Hely, 2006). Such models apply in principle not only to redundant genes, but also to binding site clusters with redundant sites and to redundant enhancers.

Redundancy is not the only route to adaptive robustness. In the context of transcriptional regulation, this became clear once it became possible to analyze the structure of genotype spaces of model transcriptional regulatory circuits. In such spaces, one finds that circuits with a given gene expression pattern usually form large and connected genotype networks, where differences between neighboring genotypes (circuits) can be caused by small genetic changes, such as alterations of single regulatory interactions (Ciliberti et al., 2007; Cotterell and Sharpe, 2010; Payne et al., 2014). Individual circuits in such a network can change their regulatory interactions without changing their expression pattern. Because these circuits also vary considerably in their mutational robustness, they can evolve increased robustness via a series of small mutations that maintain their expression phenotype. Empirical data on TF binding sites demonstrate that such sites show a similar organization in the space of DNA sequences (Payne and Wagner, 2014). In consequence, their mutational robustness could in principle increase through gradual genetic change (e.g., point mutations) that preserve transcription factor binding.

Despite these observations, robustness is unlikely to confer a sufficiently strong advantage in a binding site, regulatory circuit, or a redundant regulatory element to be maintained by natural selection in most evolving populations. The reason is that its selective advantage is small, i.e., on the order of the mutation rate μ, because selection of increased robustness is effective only when a population of organisms (binding sites, circuits, etc.) are polymorphic for robustness. Elementary population genetics dictates that this will be the case only when the product of the effective population size *N* and the mutation rate μ is much greater than one (*Nμ* ≫ 1) (van Nimwegen et al., 1999; Wagner, 2000). Especially for small mutational targets, this requires huge population sizes and very large mutation rates. Therefore, although robustness may sometimes be an adaptation, this is likely the exception rather than the rule.

Mutational robustness may also arise as a byproduct of selection for other traits, most notably robustness to environmental change (Wagner, 1997; Meiklejohn and Hartl, 2002). This is particularly relevant for transcriptional regulation, which is frought with noise, including stochastic fluctuations in signaling molecules and variable temperatures (Macneil and Walhout, 2011). Such noise can be viewed as incessant change in the molecular environment where transcriptional regulation operates. Shadow enhancers provide a useful example. As we mentioned in Section 2.3, the regulatory region of the *Drosophila* gene *snail* comprises two enhancers. Either of them is sufficient to drive wild-type gene expression patterns under normal growth conditions (Perry et al., 2010), which provides a source of mutational robustness. Under extreme temperatures, however, deletion of either of the enhancers results in aberant gene expression patterns, suggesting that the primary function of the shadow enhancer is to provide robustness to the destabilizing effects of sub-optimal temperatures, as is also the case for the two shadow enhancers associated with the *Drosophila* gene *shavenbaby* (Frankel et al., 2010). Additional support for the origin of mutational robustness as a byproduct of environmental robustness is found in model transcriptional regulatory circuits, which exhibit a positive correlation between mutational and environmental robustness (Ciliberti et al., 2007), such that selection for environmental robustness facilitates mutational robustness.

Finally, mutational robustness may also be a consequence of biophysical principles underlying transcriptional regulation, or of non-adaptive forces of genome evolution, i.e., genetic drift, mutation, and recombination.

For example, homotypic clusters of TF binding sites may evolve simply because there are more ways to build a regulatory region using many low-affinity sites than there are with few high-affinity sites (He et al., 2012). The reason is that there are many more distinct DNA sequences that bind TFs with low affinity than with high affinity (Badis et al., 2009). In addition, such clusters could simply result from the inefficiency of selection at removing insertions, such that insertions containing TF binding sites accumulate over time (Lynch, 2007), or they may be a byproduct of recombination within regulatory regions (Lynch, 2007; Paixao and Azevedo, 2010). Moreover, the spatial organization of homotypic clusters may reflect a mutational bias toward deletions, as such mutations are more likely to bring different sites closer together than farther apart (Lusk and Eisen, 2000).

Similarly, robustness-conferring topological properties, such as heavy-tailed degree distributions, can originate as a by-product of biophysical principles. For example, a biophysical model of protein-protein interactions shows that this distribution can emerge if the number of surface-exposed hydrophobic amino acids on a protein follows a simple random distribution (Deeds et al., 2006). In addition, evolutionary forces other than natural selection can enhance the robustness of regulatory networks. For instance, heavy-tailed degree distributions (Lynch, 2007), a modular organization (Wagner et al., 2007), and the enrichment of particular circuit motifs (Artzy-Randrup et al., 2004; Cordero and Hogeweg, 2006; Sorrells and Johnson, 2015) can all emerge through random genetic drift.

## 4. Consequences of robustness

Mutational robustness in transcriptional regulation has several consequences that emerge on evolutionary timescales. First, the mutational robustness of regulatory regions permits their evolutionary divergence without a corresponding divergence in the gene expression patterns they control. This phenomenon is often observed among closely-related species (Weirauch and Hughes, 2010). During such divergence, substantial binding site turnover may occur, such that different sets of TFs may regulate orthologous genes in different species (Moses et al., 2006; Borneman et al., 2007; Schmidt et al., 2010). Binding site turnover can even occur among activating and repressing TFs and can alter the architecture of a regulatory circuit, all without altering its gene expression phenotype (Tanay et al., 2005; Tsong et al., 2006; Swanson et al., 2011). A well-known practical consequence of this divergence is that regulatory regions are exceptionally difficult to align.

A related consequence of mutational robustness is that regulatory regions can accumulate genetic diversity within a population. Such diversity is often referred to as cryptic, because it does not generate phenotypic variation (Gibson and Dworkin, 2004; McGuigan and Sgro, 2009). However, cryptic diversity may generate phenotypic variation upon environmental or genetic perturbation (Rutherford and Lindquist, 1998; Queitsch et al., 2002). Cryptic diversity is commonly observed in DNA sequences regulating transcription (Rockman and Wray, 2002), including TF binding sites (Balhoff and Wray, 2005; Kasowski et al., 2010; Spivakov et al., 2012; Arbiza et al., 2013). Computational models of transcriptional regulatory circuits hint that such diversity may generate phenotypic variation in response to genetic or environmental perturbations (Siegal and Bergman, 2002; Bergman and Siegal, 2003). However, we currently have no experimental evidence that standing cryptic diversity in gene regulatory regions contributes to adaptation in transcriptional regulation.

Yet another consequence of mutational robustness is that it permits regulatory interactions to originate that do not contribute to gene regulation at the time of their origin. Over time, the accumulation of such non-functional interactions can give rise to dense, highly-interconnected transcriptional regulatory networks (Sorrells and Johnson, 2015). This is especially true if binding sites are short, regulatory regions are long, and TF binding specificities are low. Evidence exists that each of these conditions are met, especially in eukaryotes, where binding sites are on average merely ten nucleotides long (Stewart et al., 2012), regulatory regions comprise promoters and enhancers that span thousands of nucleotides (The ENCODE Project Consortium, 2012), and the average information content per nucleotide of binding sites is roughly 65% of the maximum, indicating modest specificity (Stewart et al., 2012). Taken together with evidence that synthetically-added regulatory interactions rarely impact phenotype (Isalan et al., 2008), these observations suggest that mutational robustness may contribute to the apparent complexity of transcriptional regulatory networks. What is more, non-functional regulatory interactions may form the substrate of subsequent adaptations (Isalan et al., 2008), implicating mutational robustness in the evolution of novel transcriptional regulatory programs.

A final consequence of robustness emerges from the duplication of transcription factor genes. By providing a back-up gene for any one essential molecular function, gene duplication facilitates the evolution of genes with novel functions (Ohno, 1970; Hahn, 2009; Innan and Kondrashov, 2010; Rensing, 2014), such as TFs with altered binding site repertoires that can take on novel regulatory roles (Pérez et al., 2014). Over long evolutionary time scales, this ability can have profound consequences. For example, gene and genome duplications that created novel homeobox TF genes have been implicated in the diversification of the vertebrate body plan (Carroll et al., 2001), and duplication of genes encoding MADS box TFs has played an important role in the diversification of flowering plants (De Bodt et al., 2003; Irish, 2003). In other words, robust transcriptional regulation has helped shape life as we know it.

## Author contributions

JP and AW conceived of and wrote the paper.

## Funding

JP acknowledges support through the Ambizione program of the Swiss National Science Foundation. AW acknowledges support through Swiss National Science Foundation grant 31003A_146137, as well as through the University Priority Research Program in Evolutionary Biology at the University of Zurich.

### Conflict of interest statement

The authors declare that the research was conducted in the absence of any commercial or financial relationships that could be construed as a potential conflict of interest.
